# P-1877. Daptomycin-Induced Creatine Phosphokinase Elevations Among Patients Requiring Outpatient Parenteral Antimicrobial Therapy

**DOI:** 10.1093/ofid/ofae631.2038

**Published:** 2025-01-29

**Authors:** Deanna Berg, Amanda Binkley, Christina Maguire, Anne Norris, Vasilios Athans

**Affiliations:** Penn Presbyterian Medical Center, Philadelphia, PA; Penn Presbyterian Medical Center, Philadelphia, PA; Penn Presbyterian Medical Center, Philadelphia, PA; Penn Presbyterian Medical Center, Philadelphia, PA; Hospital of the University of Pennsylvania, Philadelphia, Pennsylvania

## Abstract

**Background:**

Daptomycin, a lipopeptide with broad-spectrum gram-positive activity, requires once-daily dosing with weekly creatine phosphokinase (CPK) monitoring for daptomycin-induced muscle injury. There is a paucity of data that describes the incidence of CPK elevations among patients who require extended durations of daptomycin outpatient parenteral antimicrobial therapy (OPAT). Variables associated with elevated CPK levels include high-dose daptomycin (≥ 8 mg/kg), elevated body mass index, and concomitant medication. This study sought to describe the laboratory monitoring and safety profile of daptomycin when used in the OPAT setting.

**Figure:**
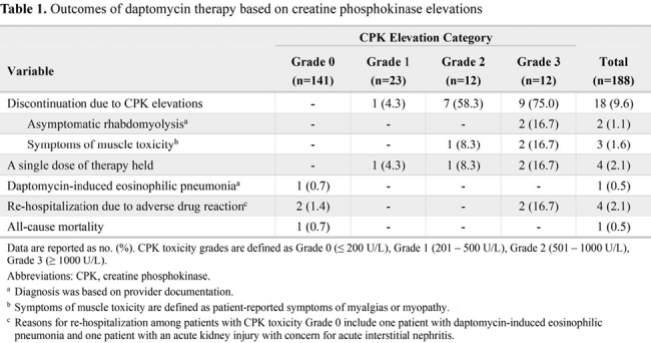

**Methods:**

We conducted a retrospective, multicenter cohort study of adults who received daptomycin OPAT for at least seven days between January 1, 2022, and December 31, 2023. Patients with an unknown or elevated baseline CPK were excluded. CPK toxicity grades are defined as Grade 0 (≤ 200 U/L), Grade 1 (201-500 U/L), Grade 2 (501-1000 U/L), Grade 3 (≥ 1000 U/L). The primary endpoint was the incidence of CPK elevations during daptomycin OPAT. Secondary endpoints include characterizing factors associated with CPK elevations and safety outcomes.

**Results:**

A total of 188 patients were included in the analysis. Most patients were treated for osteomyelitis (46%), endovascular (19%), or prosthetic joint infection (18%) caused by either staphylococci (63%) or enterococci (19%). The median weight-based daptomycin dose was 7 mg/kg (actual body weight) (IQR 6 – 8), and the median treatment duration was 38 days (IQR 24 – 42 ). A total of 86 patients were on concomitant statin therapy, of which 54% were on a high-intensity statin, and 79% were on a lipophilic statin. CPK toxicity Grade 0 was the highest toxicity among 70% of patients on statin therapy. There were 47 patients (25%) who experienced any CPK elevation (Grade ≥ 1), of which the majority were asymptomatic (44/47, 94%), and CPK elevation led to discontinuation of therapy in 18 patients (Table 1).

**Conclusion:**

The majority of the patients were able to tolerate prolonged daptomycin therapy. Concomitant statin therapy was continued in most patients. Repeat CPK testing in asymptomatic patients and assessment of confounding factors may be appropriate prior to early discontinuation of daptomycin therapy.

**Disclosures:**

Amanda Binkley, PharmD, BCIDP, AAHIVP, Viiv: Advisor/Consultant Christina Maguire, PharmD, Viiv: Advisor/Consultant

